# Practical Management of Zolbetuximab Administration: The Project VYLOY Initiative †

**DOI:** 10.3390/cancers17121996

**Published:** 2025-06-15

**Authors:** Yukiya Narita, Taro Mizuno, Takato Suda, Junko Kurono, Yasunobu Ishizuka, Yumi Iida, Akiko Kondo, Kazuhiro Shimomura, Chisato Yamada, Eri Hotta, Koji Kuraishi, Kanae Tozaki, Makiko Kobara, Chihoko Takahata, Kei Muro

**Affiliations:** 1 Department of Clinical Oncology, Aichi Cancer Center Hospital, Nagoya 464-8681, Japan; ta.mizuno@aichi-cc.jp (T.M.); y.ishizuka@aichi-cc.jp (Y.I.); kmuro@aichi-cc.jp (K.M.); 2 Department of Pharmacy, Aichi Cancer Center Hospital, Nagoya 464-8681, Japan; t.suda@aichi-cc.jp (T.S.); y.iida@aichi-cc.jp (Y.I.); a.kondo@aichi-cc.jp (A.K.); kazshimomura@aichi-cc.jp (K.S.); 3 Department of Nursing, Aichi Cancer Center Hospital, Nagoya 464-8681, Japan; j.kurono@aichi-cc.jp (J.K.); c.yamada@aichi-cc.jp (C.Y.); e.hotta@aichi-cc.jp (E.H.); k.kuraishi@aichi-cc.jp (K.K.); ctakahata@aichi-cc.jp (C.T.); 4 Medical Safety Management Office, Aichi Cancer Center Hospital, Nagoya 464-8681, Japan; k-tozaki@aichi-cc.jp; 5 Clinical Trial Department, Aichi Cancer Center Hospital, Nagoya 464-8681, Japan; m-kobara@aichi-cc.jp

**Keywords:** claudin 18.2, gastric cancer, nausea, vomiting, zolbetuximab

## Abstract

**Background:** Zolbetuximab, a monoclonal antibody targeting claudin-18.2 (CLDN18.2), which was recently approved as first-line treatment for advanced gastric cancer (AGC), presents unique safety challenges, particularly infusion-related gastrointestinal toxicity and hypoalbuminemia. This study aimed to present our experience with zolbetuximab administration in patients with AGC, focusing on the safety and management effectiveness of our adapted protocol in routine clinical practice. **Methods:** This study presents our single-institution real-world experience implementing a proactive management protocol (“Project VYLOY”) using zolbetuximab to mitigate these toxicities. We adopted a standardized stepwise infusion protocol and antiemetic premedication to reduce infusion-related nausea and vomiting. Patients with CLDN18.2-positive advanced gastric or gastroesophageal junction adenocarcinoma who received zolbetuximab combined with chemotherapy were included. **Results:** Twenty-four patients were included. The median infusion duration was 215 min, with an interruption rate of 25.0%. In cycle 1, 62.5% experienced infusion-associated adverse events, primarily grade 1 nausea (54%) and vomiting (25%). Hypoalbuminemia (grade ≥ 2) occurred in 57% of first-line patients, potentially linked to zolbetuximab-induced gastritis and gastrointestinal protein loss. Proactive antiemetic support and infusion rate adjustments substantially reduced infusion interruptions in subsequent cycles (10.9%). Patients without prior gastrectomy had higher nausea and vomiting rates, confirming the stomach’s role in mediating toxicity. **Conclusions:** Our results suggest that proactive management can improve the safety and tolerability of zolbetuximab, especially by reducing infusion-related toxicity in real-world practice.

## Simple Summary

Zolbetuximab is a new drug used to treat advanced stomach cancer that expresses a protein called CLDN18.2. While this treatment can be effective, it may cause side effects such as nausea, vomiting, and low blood protein levels, especially during the first infusion. In our hospital, we introduced a careful support plan called “Project VYLOY” to reduce these side effects. We analyzed the treatment experience of 24 patients and found that adjusting the infusion speed and giving strong anti-nausea medicine helped to improve safety and reduced problems in later treatments. Patients who had not had stomach surgery were more likely to feel sick, suggesting that the stomach plays a role in how the drug causes side effects. Our results show that by carefully managing how zolbetuximab is given, doctors can make the treatment safer and more comfortable for patients in everyday clinical settings.

## 1. Introduction

Gastric cancer (GC) is the fifth most common type of cancer and the fifth-leading cause of cancer-related mortality worldwide [1]. Although using several chemotherapeutic cytotoxic and molecular-targeted agents or immune-checkpoint inhibitors has improved survival in patients with advanced GC (AGC), the median overall survival (OS) is still suboptimal [2,3,4,5,6,7].

Zolbetuximab is a first-in-class monoclonal antibody targeting claudin-18.2 (CLDN-18.2), recently approved for use in combination with platinum and fluoropyrimidine chemotherapy as a first-line treatment for advanced gastric and gastroesophageal junction (GEJ) adenocarcinoma [7,8,9,10,11,12,13]. However, an emerging challenge with zolbetuximab is its unique safety profile, most notably a high incidence of nausea and vomiting during infusions (all-grade nausea and vomiting, 82% and 67%, respectively). These symptoms are more frequent during the first treatment cycle. In fact, early real-world experiences have echoed these observations; for example, Shimozaki et al. reported that 62% of patients required infusion interruption due to intolerable nausea in cycle 1 despite a controlled infusion rate [14]. Notably, patients without a stomach (post-gastrectomy) appear less prone to zolbetuximab-induced nausea and vomiting, suggesting that the stomach is the primary site mediating this effect.

Decreased appetite was also more frequent with zolbetuximab, and hypoalbuminemia emerged as a notable adverse event (AE) associated with zolbetuximab therapies. In the GLOW trial, any-grade hypoalbuminemia was reported in 22.4% of patients treated with zolbetuximab plus capecitabine plus oxaliplatin (CAPOX), versus 14.1% with CAPOX alone [8]. These findings suggest that beyond tumor control, zolbetuximab’s on-target effects can affect patients’ nutritional status and laboratory parameters.

Ensuring that patients safely receive the full zolbetuximab dose is critical for maximizing efficacy [15]. Recent consensus guidelines have begun to address this issue, recommending tailored infusion rate adjustments and aggressive antiemetic support [16]. Our institution launched the “Project VYLOY” initiative, which was executed by a multidisciplinary team composed of physicians, nurses, and pharmacists [17,18]. This study presents our experience with zolbetuximab administration in patients with AGC, focusing on the safety and management effectiveness of our adapted protocol in routine clinical practice.

## 2. Materials and Methods

### 2.1. Patients and Treatment

This single-institution observational study was conducted on patients treated with zolbetuximab as part of standard care for advanced gastric/GEJ cancer. We consecutively included all patients who started zolbetuximab between 19 July 2024 and 10 February 2025. Eligibility for zolbetuximab required immunohistochemical confirmation of a CLDN18.2-positive tumor (per regulatory approval criteria) and advanced, human epidermal growth factor receptor 2-negative gastric or GEJ adenocarcinoma [10,19,20]. Patients received zolbetuximab in combination with a fluoropyrimidine–platinum doublet regimen of the treating physician’s choice.

Zolbetuximab was administered via intravenous (IV) infusion on day 1 of each chemotherapy cycle (every 2 or 3 weeks depending on the regimen). The cycle 1 loading dose was 800 mg/m^2^, and subsequent doses were 600 or 400 mg/m^2^ (for 3-week or 2-week cycles).

### 2.2. Stepwise Infusion Protocol

Zolbetuximab was reconstituted and diluted in normal saline to achieve a total infusion volume of 600 or 320 mL, depending on the prescribed dose (Table 1). A stepwise infusion rate escalation was used to mitigate infusion-related nausea/vomiting and other potential reactions. Infusions began at level (Lv) 1, corresponding to 100 mg/m^2^/h. If no moderate or severe symptoms arose, the infusion rate gradually increased (to Lv2, Lv3, and Lv4) at predefined intervals until the full dose was delivered. From cycle 2 onward, the infusion began at the same final rate used in the previous cycle, provided that the patient had tolerated that rate without grade ≥2 toxicity. If the patient had experienced infusion-related AEs, the starting level could be lowered at the physician’s discretion.

### 2.3. Management of Nausea/Vomiting and Infusion Interruptions

**Figure 1:**
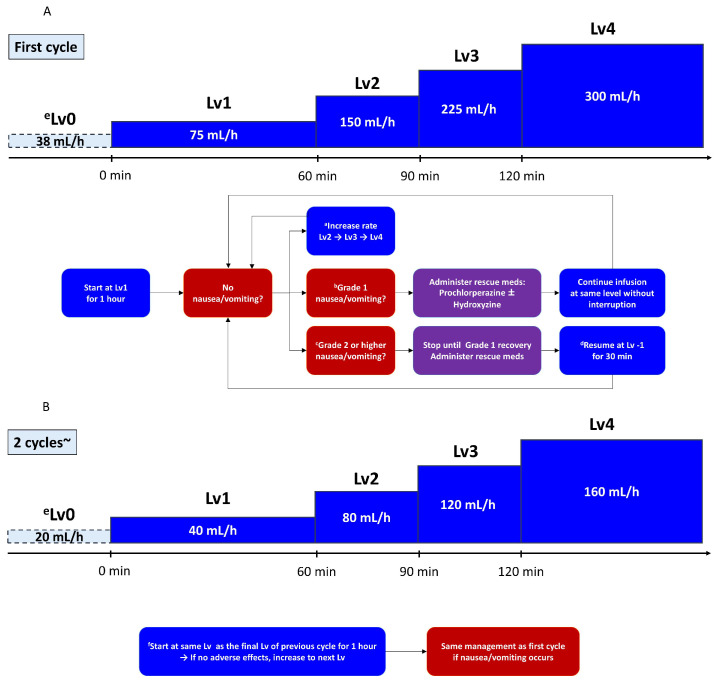
Protocol decision flow for zolbetuximab infusion: (**A**) initial (first) cycle; (**B**) subsequent cycles (from cycle 2 onward). ^a^ If no adverse events occurred, increase the rate after 1 h; ^b^ in the case of mild nausea or vomiting, administer antiemetics immediately and continue infusion without interruption; ^c^ for grade ≥ 2 nausea or vomiting, suspend infusion; ^d^ once symptoms improved, resume at one level lower (Lv−1); ^e^ if infusion is suspended at Lv1, resume at Lv0 (minimum rate); ^f^ patients begin at the same infusion level (Lv) as the final Lv of the previous cycle and remain at that rate for 1 h.

In cases of mild (grade 1 according to CTCAE v5.0 [21].) nausea/vomiting, the infusion was continued without interruption; however, additional antiemetics (e.g., IV prochlorperazine or hydroxyzine) were administered promptly (Figure 1).

For moderate to severe (grade ≥ 2) nausea/vomiting, the infusion was immediately paused, and rescue antiemetics was given. Once symptoms improved to grade ≤1, the infusion was resumed at Lv–1 (one level below the rate at which the event occurred). If the patient had been at Lv1 when the AE arose, the infusion was resumed at Lv0 (the minimum flow rate) defined as half the infusion rate of Lv1; however, the infusion rate was not lowered to below Lv0.

In subsequent cycles, patients were generally started at the final tolerated rate in the prior cycle. If the patient had experienced grade ≥2 toxicity in the previous cycle, the physician could opt for a slower start (e.g., beginning again at Lv1) with gradual escalation.

All patients were premedicated with a standard antiemetic regimen consisting of a 5-hydroxytryptamine (serotonin) receptor type 3 (5-HT3) receptor antagonist (granisetron), a neurokinin-1 (NK1) receptor antagonist (fosnetupitant), and dexamethasone before zolbetuximab infusion (Figure S1) [22,23,24,25,26,27]. In addition to antiemetic agents, all patients received a potassium competitive acid blocker (vonoprazan) and rebamipide for gastric mucosal protection during the treatment course. Additional agents such as olanzapine were used at the oncologist’s discretion in patients with refractory nausea in subsequent cycles. During infusion, patients were carefully monitored for vital sign changes and symptoms. Nurses were ready with rescue medications (e.g., IV metoclopramide and diphenhydramine) at the bedside.

### 2.4. Data Collection

Patients were evaluated before each treatment cycle through physical examination, performance status assessment, laboratory tests, and toxicity review. Serum albumin was specifically measured at baseline (within 1 week before the first zolbetuximab dose) and before each subsequent cycle. Albumin values were recorded from the start of treatment or until treatment discontinuation, whichever occurred first. For the analysis of albumin kinetics, the analysis focused on 14 patients who received zolbetuximab in the first-line setting to evaluate albumin changes over time in previously untreated patients.

Infusion start/end times, interruptions (yes/no and duration), and any AEs occurred during or immediately after each zolbetuximab infusion were prospectively recorded. Nausea and vomiting severity was graded according to CTCAE v5.0 [21]. The total infusion time was defined as the total time elapsed from the start to the end of the zolbetuximab infusion, including any interruptions.

### 2.5. Statistical Analysis

All eligible patients treated during the study period were consecutively included. Descriptive statistics were used to summarize patient demographics, treatment exposure, and safety outcomes. Categorical variables were presented as frequencies and percentages, and continuous variables were expressed as medians with ranges or means with standard deviations, as appropriate. The normality of continuous data distributions was assessed using the Shapiro–Wilk test. Given the observational nature of the study and small sample size, no comparative or inferential statistical tests (e.g., *t*-test or chi-square test) were performed. Missing data were handled by complete case analysis. Tumor response was assessed by imaging and/or endoscopy per routine care and categorized using Response Evaluation Criteria in Solid Tumors (RECIST) 1.1 criteria, when available (complete response, partial response, stable disease, or progressive disease) [28]. No formal hypothesis testing was performed given the small sample size and single-arm nature of the study. The patients were not stratified or compared by subgroups. Progression-free survival (PFS) was defined as the interval from the first dose of zolbetuximab to the date of documented disease progression or death from any cause. The data cutoff date was 4 April 2025. R software version 4.1.0 (R Project for Statistical Computing, Vienna, Austria) was used to carry out the statistical analyses.

## 3. Results

### 3.1. Patient Characteristics and Treatment Exposure

Twenty-four patients were included in the analysis (Table 2). The median age was 67 (range, 41–81) years, with 16 men (67%) and 8 females (33%). The Eastern Cooperative Oncology Group Performance Status (ECOG PS) was 0 in 15 patients (63%), 1 in 7 patients (29%), and 2 in 2 patients (8%). Eight patients (33%) had undergone prior gastrectomy (distal gastrectomy in three, total gastrectomy in three, and proximal gastrectomy in two). The primary tumor was located in the stomach in 21 (88%) cases and at the EGJ in 3 (12%). Histologically, 21 (88%) were diffuse-type adenocarcinoma, and 3 (13%) were intestinal-type adenocarcinoma. Peritoneal metastases were present in 22 (92%) patients, and other common metastatic sites included lymph nodes (50%), the liver (21%), and lungs (8%). Fourteen patients (58%) received zolbetuximab as first-line therapy, whereas the remaining ten (42%) were treated with second-line therapy or beyond. The median time from the diagnosis of advanced disease to zolbetuximab initiation was 1.5 months for first-line cases and 11 months for later-line cases.

Regarding treatment exposure, patients received a total of 193 cycles of zolbetuximab (median, 4 cycles per patient; range, 1–14). At the endpoint of the study, 11 (46%) patients were still on treatment, and 1 (4%) was lost to follow-up. Of the 12 patients who discontinued treatment, 11 (92%) did so due to radiologic progression. The remaining patient died from aspiration pneumonia with acute respiratory distress syndrome, precluding further therapy.

Chemotherapy backbones included l-leucovorin (l-LV), 5-fluorouracil (5-FU), and oxaliplatin (FOLFOX) in 11 (46%) patients, CAPOX in 5 (21%), and S1 plus oxaliplatin (SOX) in 4 (17%). Two patients (8%) received an infusion of 5-FU/l-LV with zolbetuximab, and 2 (8%) received zolbetuximab alone. Among first-line cases, CAPOX (43%) and SOX (29%) were common.

### 3.2. Acute Infusion-Related Tolerability

**Figure 2:**
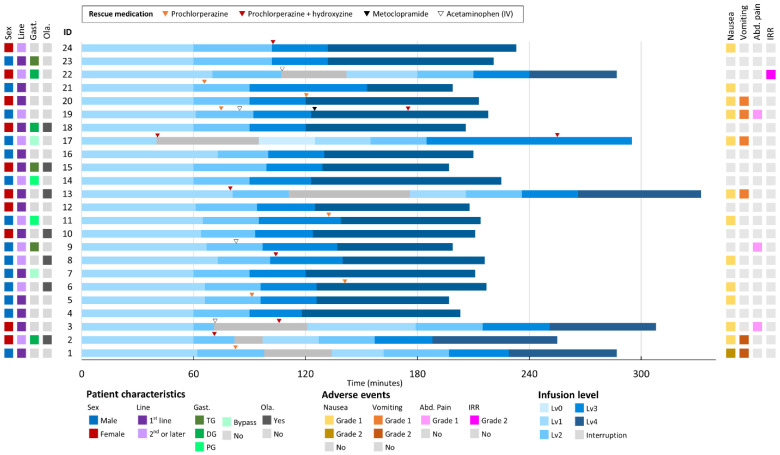
Overview for the first zolbetuximab infusion. Abbreviations: Abd. Pain, abdominal pain; Gast., gastrectomy; IRR, infusion-related reaction; Line, treatment line; Ola., olanzapine use.

In cycle 1, 15 of 24 (63%) patients experienced infusion-associated AEs, primarily nausea (54%) and vomiting (25%), with most cases being grade 1 (Figure 2). Two patients experienced multiple episodes of emesis during the first 1–2 h despite prophylaxis, and received additional IV antiemetics before resuming at a slower rate. One patient had intense nausea and epigastric pain for approximately 90 min. This was managed with a pause, IV prochlorperazine and acetaminophen, and a restart at half the initial rate.

Importantly, after patients 1–3 experienced consecutive infusion interruptions, our institution adjusted the protocol by ensuring that rescue medications were readily available at the bedside and by instructing patients to immediately report even mild nausea. This proactive approach—prompting nursing staff to administer rescue antiemetics at the first indication of discomfort—resulted in significantly fewer interruptions in subsequent infusions. Following these changes, no further grade ≥ 2 vomiting occurred during infusion.

Patients who have a history of gastrectomy tended to have less frequent nausea and vomiting. Among 16 patients who have no history of gastrectomy, 11 (69%) had nausea and vomiting during cycle 1, whereas only 2 of 8 (25%) patients with prior gastrectomy experienced nausea and vomiting during infusion.

### 3.3. Infusion Time and Infusion Interruption Rates

In cycle 1, total infusion times had a median of 215 min (range, 197–332) and a mean of 232 ± 40 min (Table 3). In contrast, for subsequent cycles (cycles 2 and beyond), the median infusion time was 131 (range, 42–296) min and the mean was 140 ± 35 min. For cycle 1, 25.0% of the patients experienced at least one interruption during infusion. In contrast, for cycles 2 and beyond, the interruption rate was 10.9%. During cycle 1, the median time to interruption and interruption duration were 90 (range, 40–111) and 43 (range, 15–65) min, respectively. In contrast, for cycles 2 and beyond, the median time to interruption and interruption duration decreased to 71 and 28 min, respectively. Nine patients did not experience any infusion-related AEs during cycle 1. Among them, such AEs occurred in four (44%) patients during subsequent cycles. These AEs first occurred in cycle 2 or 3.

### 3.4. Postinfusion and Delayed AEs

During the interval between zolbetuximab infusions, the most common delayed toxicities were nausea (39%), anorexia (26%), and fatigue (18%) (Figure S2, Table S1). Grade ≥ 2 anorexia was observed in 13% of the patients, including 3% with grade 3 severity.

### 3.5. Serum Albumin Kinetics and Hypoalbuminemia

In the first-line group (14 patients), the median baseline albumin was 3.8 (range, 2.3–4.6) g/dL, and serum albumin levels declined in all but one patient as therapy progressed (Figure S3). Moreover, grade 2 hypoalbuminemia developed in 57% of the patients, and grade 3 developed at some point during first-line treatment in 7%. In cases where zolbetuximab was temporarily discontinued because of severe appetite loss or other reasons, hypoalbuminemia rapidly improved by the start of the next cycle following treatment resumption. Gastric mucosal inflammation suspected to be zolbetuximab-related was observed after three cycles in a patient with CLDN18.2-positive type 4 gastric cancer (Figure S4). Owing to the limited number of patients and events in the first-line subgroup, detailed efficacy data are presented in the Supplementary Materials.

## 4. Discussion

This study constitutes the most extensive real-world dataset to date on patients treated with zolbetuximab for advanced CLDN18.2-positive gastric or GEJ cancer. Our findings highlight the critical role of proactive safety management, particularly regarding infusion-related gastrointestinal toxicity and hypoalbuminemia, which significantly influence treatment tolerability and continuity.

We observed that infusion-related nausea and vomiting were influenced by patient background factors, notably prior gastrectomy (they occurred in 69% of patients without prior gastrectomy and 25% of patients with prior gastrectomy). This finding aligns closely with the pivotal SPOTLIGHT and GLOW trials, which reported higher nausea (72–76%) and vomiting (72–76%) rates in patients without prior gastrectomy than those with gastrectomy (nausea, 48–59%; vomiting, 48–52%) [7,8]. These findings confirm the stomach as a primary site mediating zolbetuximab-related gastrointestinal toxicities, underscoring the need for individualized prophylactic strategies based on surgical history.

This study demonstrated marked improvement in infusion tolerability after cycle 1. The infusion interruption rate decreased substantially from 25% in cycle 1 to 10.9% in subsequent cycles, and the median interruption duration decreased from 43 to 28 min. To our knowledge, this is the first detailed real-world study showing such clear improvement in infusion-related tolerability after the initial administration of zolbetuximab, highlighting the importance of careful management strategies during the first infusion. This improvement aligns with the consensus guidelines, which advocate for aggressive prophylactic antiemetic regimens that include NK1 receptor antagonists, 5-HT3 receptor antagonists, dexamethasone, and olanzapine, as well as a stepwise infusion rate escalation strategy with immediate pauses for infusion it symptoms arise [16]. Our protocol adhered to these guidelines but was distinctively proactive, involving immediate bedside availability of rescue medications and early patient-initiated reporting of mild symptoms.

A notable finding was the emergence of infusion-related AEs in subsequent cycles among patients who did not initially experience these toxicities, with 44% during cycles 2 or 3. This contrasts sharply with findings from the SPOTLIGHT and GLOW trials, where nausea and vomiting consistently decreased with subsequent cycles [7,8]. This discrepancy may be attributed to differences in antiemetic protocols, patient selection, and real-world adherence to supportive care. In contrast to clinical trials, patients in our cohort may have experienced delayed gastric inflammation due to cumulative mucosal injury or variability in the implementation of antiemetic strategies across cycles. Thus, our results suggest the necessity of ongoing vigilant monitoring and proactive management even after the initial successful administration, which may be overlooked based on previous clinical trial patterns.

Hypoalbuminemia emerged as a significant concern, with a markedly higher incidence (grade ≥ 2.57%) than reported in SPOTLIGHT and GLOW (16–18%) [7,8]. The temporal association with gastrointestinal symptoms, supported by recent real-world endoscopic evidence, suggests that zolbetuximab-induced gastritis and potential protein-losing gastroenteropathy play critical roles in the albumin decline [29,30,31]. Nutritional interventions alone may be insufficient for severe hypoalbuminemia. Temporary zolbetuximab treatment interruption should be considered in patients experiencing significant albumin depletion.

Comparative analysis with the existing literature, particularly Shimozaki et al., revealed that our median infusion duration of 215 min and interruption rate of 25% in cycle 1 were lower than their reported 257 min and 62%, respectively [14]. These improvements likely reflect the effect of proactive supportive care, including timely antiemetic use and infusion rate adjustments. Given that frequent interruptions of zolbetuximab naturally prolong the total infusion time and that backbone cytotoxic regimens often require additional time, a higher interruption rate can substantially increase patients’ time toxicity [32]. Minimizing such burdens is essential for enhancing the overall treatment experience and patient quality of life.

This study has several limitations to acknowledge, including its retrospective single-institution design, relatively small sample size, and short median follow-up duration, which limits the robustness of efficacy outcomes and generalizability. In addition, variations in supportive care practices across institutions may influence the broader applicability of our findings. We encourage the conduction of large sample size prospective multicenter studies to further zolbetuximab-related AEs and contributing factors.

## 5. Conclusions

Our real-world experience emphasizes the importance of effective management of zolbetuximab-associated gastrointestinal toxicity and hypoalbuminemia, informed by patient background and cycle-specific tolerability patterns. This proactive, patient-centered approach facilitates treatment adherence and maximizes potential therapeutic outcomes.

## Figures and Tables

**Table 1:** Zolbetuximab preparation and administration.

Dosage	800 mg/m^2^	600 mg/m^2^	400 mg/m^2^
**Preparation Method**	Total volume: 600 mL	Total volume: 320 mL	Total volume: 320 mL
**Infusion Rate**			
**Lv1 (100 mg/m^2^/h)**	75 mL/h	40 mL/h	40 mL/h
**Lv2 (200 mg/m^2^/h)**	150 mL/h	80 mL/h	80 mL/h
**Lv3 (300 mg/m^2^/h)**	225 mL/h	120 mL/h	120 mL/h
**Lv4 (400 mg/m^2^/h)**	300 mL/h	160 mL/h	160 mL/h
**Lv0 (50 mg/m^2^/h)**	38 mL/h	20 mL/h	20 mL/h

Example for BSA 1.5 m^2^: 800 mg/m^2^ = 1200 mg (60 mL at 20 mg/mL). Infusion: 500 mL saline + 60 mL zolbetuximab + 40 mL saline (flush).

**Table 2:** Patient characteristics.

Characteristic		*N* = 24 (%)
Age, years	Median (range)	67 (41–81)
Sex	Male	16 (67)
	Female	8 (33)
ECOG PS	0	15 (63)
	1	7 (29)
	2	2 (8)
Histology	Intestinal	3 (13)
	Diffuse	21 (88)
Location of primary tumor	EGJ	3 (13)
	Stomach	21 (88)
Prior gastrectomy	Total gastrectomy	3 (12)
	Distal gastrectomy	3 (12)
	Proximal gastrectomy	2 (8)
	Bypass	2 (8)
	No	14 (58)
Disease status	Metastatic	16 (67)
	Recurrent	8 (33)
Metastatic sites	Liver	5 (21)
	Lung	2 (8)
	Peritoneum	22 (92)
	Lymph node	12 (50)
Ascites *	Massive	4 (17)
	Moderate	4 (17)
	Mild	6 (25)
	None	10 (42)
PD-L1 CPS (28–8 or 22C3)	<1	6 (25)
	1–4	3 (12)
	1–9	5 (21)
	≥5	2 (8)
	≥10	5 (21)
	NA	3 (12)
MSI-H/dMMR status	MSI-H/dMMR	0
	MSS/pMMR	17 (71)
	NA	7 (29)
Line of therapy	First-line	14 (58)
	≥Second-line	10 (42)
Backbone chemotherapy regimen	CAPOX	5 (21)
	SOX	4 (17)
	FOLFOX	11 (46)
	5-FU/l-LV	2 (8)
	Zolbetuximab monotherapy	2 (8)
Serum albumin, g/dL **	Median (range)	3.8 (2.3–4.6)

*: Massive ascites was defined as extending throughout the entire abdominal cavity; moderate ascites was defined as inconsistent with either mild or massive ascites; mild ascites was defined as localized only in the upper or lower part of the abdominal cavity; no ascites was defined as undetectable by computed tomography scanning. **: Only among first-line patients. Abbreviations: EGJ, esophago–gastric junction; ECOG, Eastern Cooperative Oncology Group; PS, performance status; CAPOX, capecitabine plus oxaliplatin; FOLFOX, l-leucovorin, 5-fluorouracil, and oxaliplatin; SOX, S-1 plus oxaliplatin; 5-FU/l-LV, 5-fluorouracil/l-leucovorin; PD-L1, programmed death-ligand 1; CPS, combined positive score; NA, not available; MSI-H, microsatellite instability–high; MSS, microsatellite stable; dMMR, deficient mismatch repair; pMMR, proficient mismatch repair.

**Table 3:** Infusion time and infusion interruption.

		Cycle 1	Cycle 2 and Beyond
Interruption rate		25.0%	10.9%
Time to interruption, min	median (range)	90 (40–111)	71 (19–125)
	mean ± SD	85 ± 27	71 ± 32
Interruption duration, min	median (range)	43 (15–65)	28 (0–111)
	mean ± SD	43 ± 18	41 ± 32
Total infusion time, min	median (range)	215 (197–332)	131 (42–296)
	mean ± SD	232 ± 40	140 ± 35
Actual infusion time, min	median (range)	215 (197–267)	130 (42–296)
	mean ± SD	222 ± 20	135 ± 29

Abbreviation: min, minutes; SD, standard deviation.

## Data Availability

The data presented in this study are available in the article and Supplementary Materials.

## Supplementary Materials

The following supporting information can be downloaded at: https://www.mdpi.com/article/10.3390/cancers17121996/s1, Figure S1. Premedication and rescue antiemetic protocol for zolbetuximab therapy. Visual summary of the standard antiemetic regimen used at our institution. Mandatory medications (◯) included a 5-HT3 receptor antagonist (granisetron), an NK1 receptor antagonist (fosnetupitant), and dexamethasone. Optional agents (△), such as olanzapine, were added based on physician discretion, particularly in patients with a history of refractory nausea. Rescue medications (★), including prochlorperazine, hydroxyzine, and metoclopramide, were prepared at the bedside for immediate use upon symptom onset during infusion. Figure S2. (a) Nausea and (b) vomiting during intervals. Distribution of severity grades for delayed nausea and vomiting experienced between zolbetuximab infusions. Figure S3. Longitudinal changes in serum albumin among first-line patients. Serial measurements of serum albumin in patients receiving zolbetuximab as first-line therapy (n = 14). Albumin levels declined in nearly all patients during treatment, and grade ≥ 2 hypoalbuminemia developed in 57%. Temporary improvement in albumin was seen after drug interruption in some cases. Figure S4. Endoscopic Images Before and After Zolbetuximab Treatment. Table S1. Summary Table of Selected Adverse Events Related to Zolbetuximab

## Abbreviations

The following abbreviations are used in this manuscript:

5-FU	5-fluorouracil
AEs	Adverse events
AGC	Advanced gastric cancer
CAPOX	Capecitabine plus oxaliplatin
CLDN18.2	Claudin-18.2
CTCAE	Common Terminology Criteria for Adverse Events
ECOG PS	Eastern Cooperative Oncology Group Performance Status
ESMO	European Society for Medical Oncology
FOLFOX	l-leucovorin, fluorouracil, and oxaliplatin
GEJ	Gastroesophageal junction
HER2	Human epidermal growth factor receptor 2
IV	Intravenous
NK1	Neurokinin-1
l-LV	l-leucovorin
Lv	Level
OS	Overall survival
PFS	Progression-free survival
PR	Partial response
RECIST	Response Evaluation Criteria in Solid Tumors
SOX	S-1 plus oxaliplatin
5-HT3	5-hydroxytryptamine (serotonin) receptor type 3

## References

[1] Bray F., Laversanne M., Sung H., Ferlay J., Siegel R.L., Soerjomataram I., Jemal A. (2024). Global cancer statistics 2022: GLOBOCAN estimates of incidence and mortality worldwide for 36 cancers in 185 countries. CA A Cancer J. Clin..

[2] Murad A.M., Santiago F.F., Petroianu A., Rocha P.R., Rodrigues M.A., Rausch M. (1993). Modified therapy with 5-fluorouracil, doxorubicin, and methotrexate in advanced gastric cancer. Cancer.

[3] Bang Y.J., Van Cutsem E., Feyereislova A., Chung H.C., Shen L., Sawaki A., Lordick F., Ohtsu A., Omuro Y., Satoh T. (2010). Trastuzumab in combination with chemotherapy versus chemotherapy alone for treatment of HER2-positive advanced gastric or gastro-oesophageal junction cancer (ToGA): A phase 3, open-label, randomised controlled trial. Lancet.

[4] Shitara K., Van Cutsem E., Bang Y.J., Fuchs C., Wyrwicz L., Lee K.W., Kudaba I., Garrido M., Chung H.C., Lee J. (2020). Efficacy and Safety of Pembrolizumab or Pembrolizumab Plus Chemotherapy vs Chemotherapy Alone for Patients With First-line, Advanced Gastric Cancer: The KEYNOTE-062 Phase 3 Randomized Clinical Trial. JAMA Oncol..

[5] Janjigian Y.Y., Shitara K., Moehler M., Garrido M., Salman P., Shen L., Wyrwicz L., Yamaguchi K., Skoczylas T., Campos Bragagnoli A. (2021). First-line nivolumab plus chemotherapy versus chemotherapy alone for advanced gastric, gastro-oesophageal junction, and oesophageal adenocarcinoma (CheckMate 649): A randomised, open-label, phase 3 trial. Lancet.

[6] Shitara K., Ajani J.A., Moehler M., Garrido M., Gallardo C., Shen L., Yamaguchi K., Wyrwicz L., Skoczylas T., Bragagnoli A.C. (2022). Nivolumab plus chemotherapy or ipilimumab in gastro-oesophageal cancer. Nature.

[7] Shitara K., Lordick F., Bang Y.J., Enzinger P., Ilson D., Shah M.A., Van Cutsem E., Xu R.H., Aprile G., Xu J. (2023). Zolbetuximab plus mFOLFOX6 in patients with CLDN18.2-positive, HER2-negative, untreated, locally advanced unresectable or metastatic gastric or gastro-oesophageal junction adenocarcinoma (SPOTLIGHT): A multicentre, randomised, double-blind, phase 3 trial. Lancet.

[8] Shah M.A., Shitara K., Ajani J.A., Bang Y.J., Enzinger P., Ilson D., Lordick F., Van Cutsem E., Gallego Plazas J., Huang J. (2023). Zolbetuximab plus CAPOX in CLDN18.2-positive gastric or gastroesophageal junction adenocarcinoma: The randomized, phase 3 GLOW trial. Nat. Med..

[9] Kubota Y., Kawazoe A., Mishima S., Nakamura Y., Kotani D., Kuboki Y., Bando H., Kojima T., Doi T., Yoshino T. (2023). Comprehensive clinical and molecular characterization of claudin 18.2 expression in advanced gastric or gastroesophageal junction cancer. ESMO Open.

[10] Nakayama I., Qi C., Chen Y., Nakamura Y., Shen L., Shitara K. (2024). Claudin 18.2 as a novel therapeutic target. Nat Rev Clin Oncol.

[11] Jia K., Chen Y., Sun Y., Hu Y., Jiao L., Ma J., Yuan J., Qi C., Li Y., Gong J. (2022). Multiplex immunohistochemistry defines the tumor immune microenvironment and immunotherapeutic outcome in CLDN18.2-positive gastric cancer. BMC Med..

[12] Sahin U., Türeci Ö., Manikhas G., Lordick F., Rusyn A., Vynnychenko I., Dudov A., Bazin I., Bondarenko I., Melichar B. (2021). FAST: A randomised phase II study of zolbetuximab (IMAB362) plus EOX versus EOX alone for first-line treatment of advanced CLDN18.2-positive gastric and gastro-oesophageal adenocarcinoma. Ann. Oncol. Off. J. Eur. Soc. Med. Oncol. ESMO.

[13] Angerilli V., Callegarin M., Govoni I., De Lisi G., Paudice M., Fugazzola P., Vanoli A., Parente P., Bergamo F., Luchini C. (2025). Heterogeneity of predictive biomarker expression in gastric and esophago-gastric junction carcinoma with peritoneal dissemination. Gastric Cancer Off. J. Int. Gastric Cancer Assoc. Jpn. Gastric Cancer Assoc..

[14] Shimozaki K., Ooki A., Yamahata Y., Aoyama T., Yoshino K., Tamba M., Udagawa S., Fukuoka S., Osumi H., Wakatsuki T. (2025). Managing zolbetuximab-induced nausea and vomiting: A proposal for a pragmatic approach in clinical practice. ESMO Gastrointest. Oncol..

[15] Shitara K., Cutsem E.V., Lordick F., Enzinger P.C., Ilson D.H., Shah M.A., Xu R.-H., Lonardi S., Yamaguchi K., Hung Y.-P. (2024). Final overall survival results from phase 3 SPOTLIGHT study evaluating zolbetuximab + mFOLFOX6 as first-line (1L) treatment for patients (pts) with claudin 18 isoform 2 (CLDN18.2)+, HER2−, locally advanced (LA) unresectable or metastatic gastric or gastroesophageal junction (mG/GEJ) adenocarcinoma. J. Clin. Oncol..

[16] Klempner S.J., Pazo-Cid R.A., Lonardi S., Swanson L., Arango M.J., Enzinger P., Ko A.H., Vaccaro G.M., Yamaguchi K., Saeed A. (2025). Consensus guidance for prevention and management of nausea and vomiting in patients treated with zolbetuximab + chemotherapy: A RAND/UCLA modified Delphi panel study. ESMO Gastrointest. Oncol..

[17] Yamada C., Narita Y., Suda T. Multidisciplinary Collaboration in the Clinical Implementation of Zolbetuximab Leveraging the Clinical Path. Proceedings of the 22nd Annual Meeting of Japanese Society of Medical Oncology..

[18] Suda T., Narita Y., Yamada C. Importance of Multidisciplinary Collaboration and Information Sharing in the Clinical Implementation of Zolbetuximab. Proceedings of the 97th Annual Meeting of the Japanese Gastric Cancer Association.

[19] Kubota Y., Shitara K. (2024). Zolbetuximab for Claudin18.2-positive gastric or gastroesophageal junction cancer. Ther. Adv. Med. Oncol..

[20] Rohde C., Yamaguchi R., Mukhina S., Sahin U., Itoh K., Türeci Ö. (2019). Comparison of Claudin 18.2 expression in primary tumors and lymph node metastases in Japanese patients with gastric adenocarcinoma. Jpn. J. Clin. Oncol..

[21] Institute N.C. Common Terminology Criteria for Adverse Events (CTCAE) v5.0. https://ctep.cancer.gov/protocoldevelopment/electronic_applications/ctc.htm.

[22] Kennedy S.K.F., Goodall S., Lee S.F., DeAngelis C., Jocko A., Charbonneau F., Wang K., Pasetka M., Ko Y.J., Wong H.C.Y. (2024). 2020 ASCO, 2023 NCCN, 2023 MASCC/ESMO, and 2019 CCO: A comparison of antiemetic guidelines for the treatment of chemotherapy-induced nausea and vomiting in cancer patients. Support. Care Cancer Off. J. Multinatl. Assoc. Support. Care Cancer.

[23] Razvi Y., Chan S., McFarlane T., McKenzie E., Zaki P., DeAngelis C., Pidduck W., Bushehri A., Chow E., Jerzak K.J. (2019). ASCO, NCCN, MASCC/ESMO: A comparison of antiemetic guidelines for the treatment of chemotherapy-induced nausea and vomiting in adult patients. Support. Care Cancer Off. J. Multinatl. Assoc. Support. Care Cancer.

[24] Herrstedt J., Clark-Snow R., Ruhlmann C.H., Molassiotis A., Olver I., Rapoport B.L., Aapro M., Dennis K., Hesketh P.J., Navari R.M. (2024). 2023 MASCC and ESMO guideline update for the prevention of chemotherapy- and radiotherapy-induced nausea and vomiting. ESMO Open.

[25] Kobayashi M., Kako J., Iba A., Okuyama A., Ozawa K., Abe M., Wada M., Akechi T., Iihara H., Imamura C.K. (2024). Non-pharmacological treatments for anticipatory nausea and vomiting during chemotherapy: A systematic review and meta-analysis of the Clinical Practice Guidelines for Antiemesis 2023. Int. J. Clin. Oncol. Jpn. Soc. Clin. Oncol..

[26] Murakami M., Miyata Y., Nakashima K., Abe M., Nishimura J., Wada M., Iino K., Akechi T., Iihara H., Imamura C.K. (2025). Clinical benefits of adding olanzapine to 5-HT(3) receptor antagonist, NK(1) receptor antagonist, and dexamethasone for the prevention of nausea and vomiting in highly emetogenic chemotherapy: A systematic review and meta-analysis of the Clinical Practice Guidelines for Antiemesis 2023 from the Japan Society of Clinical Oncology. Int. J. Clin. Oncol. Jpn. Soc. Clin. Oncol..

[27] Nakashima K., Yokomizo A., Murakami M., Okita K., Wada M., Iino K., Akechi T., Iihara H., Imamura C.K., Okuyama A. (2024). Efficacy and safety of dexamethasone sparing for the prevention of nausea and vomiting associated with moderately emetogenic chemotherapy: A systematic review and meta-analysis of Clinical Practice Guidelines for Antiemesis 2023 from Japan Society of Clinical Oncology. Int. J. Clin. Oncol. Jpn. Soc. Clin. Oncol..

[28] Eisenhauer E.A., Therasse P., Bogaerts J., Schwartz L.H., Sargent D., Ford R., Dancey J., Arbuck S., Gwyther S., Mooney M. (2009). New response evaluation criteria in solid tumours: Revised RECIST guideline (version 1.1). Eur. J. Cancer.

[29] Kinugasa F., Kajikawa S., Weng J., Ugawa T., Fushiki H., Yamanaka Y., Nagata M., Haggerty G., Akuzawa S., Nakazawa T. (2024). Effect of antiemetics on zolbetuximab-induced gastric injury and emesis in ferrets. J. Pharmacol. Sci..

[30] Sugiyama Y., Tanabe H., Tachibana S., Iribe K., Yuzawa S., Iwaki H., Yoshida Y., Fujiya M. (2025). Zolbetuximab-related gastritis: A case report of the patient with prolonged gastrointestinal symptoms. Gastric Cancer Off. J. Int. Gastric Cancer Assoc. Jpn. Gastric Cancer Assoc..

[31] Yanagimoto Y., Yamamoto K., Hara K., Masuike Y., Ushimaru Y., Kitamura M., Honma K., Matsuura N., Sugase T., Kanemura T. (2025). Two cases of protein-losing enteropathy induced by zolbetuximab in patients with unresectable advanced gastric cancer. Jpn. J. Clin. Oncol..

[32] Gupta A., Eisenhauer E.A., Booth C.M. (2022). The Time Toxicity of Cancer Treatment. J. Clin. Oncol. Off. J. Am. Soc. Clin. Oncol..

