# Supplementary material for: Practical Management of Zolbetuximab Administration: The Project VYLOY Initiative
Source: Cancers (Basel). 2025 Jun 15;17(12):1996. doi: 10.3390/cancers17121996 (PMC12190778; doi:10.3390/cancers17121996)
Supplement: Supplementary file 1 [file cancers-17-01996-s001.zip › Supplementary Material.pdf]

Supplementary Results: Clinical Efficacy

Median follow-up time were 5.6 months overall and 5.8 months in first-line patients. Among the 14 patients who received zolbetuximab as first-line therapy, 3 patients had measurable lesions. Of these, 1 achieved a confirmed PR, 1 had PD, and 1 was not evaluable due to short treatment duration. Among the 11 patients without measurable lesions, 8 were classified as non-CR/non-PD, 2 experienced PD, and 1 was not evaluable due to treatment related death. The median PFS in this first-line subgroup was 4.3 months. OS was not formally analyzed in this study due to the limited duration of follow-up and the small number of observed death events at the time of data cut-off.
