# Supplementary material for: Practical Management of Zolbetuximab Administration: The Project VYLOY Initiative
Source: Cancers (Basel). 2025 Jun 15;17(12):1996. doi: 10.3390/cancers17121996 (PMC12190778; doi:10.3390/cancers17121996)
Supplement: Supplementary file 1 [file cancers-17-01996-s001.zip › Supplementary Table S1.pdf]

Supplementary Table S1. Summary Table of Selected Adverse Events Related to Zolbetuximab

**Toxicity Category**	**Cycle 1**				**Cycle 2 and beyond**			
	**Any grade**	**Grade 1**	**Grade 2**	**≥ Grade 3**	**Any grade**	**Grade 1**	**Grade 2**	**≥ Grade 3**
**Acute Infusion-Related AEs**								
Nausea	54%	50%	4%	0%	23%	17%	6%	0%
Vomiting	25%	17%	8%	0%	3%	3%	0%	0%
**Post-infusion/delayed AEs**								
Nausea	58%	46%	8%	4%	23%	17%	6%	0%
Vomiting	0%	0%	0%	0%	3%	3%	0%	0%
Anorexia	62%	21%	17%	25%	32%	14%	18%	0%
Fatigue	21%	12%	8%	0%	32%	27%	0%	5%
Diarrhea	8%	4%	4%	0%	18%	9%	9%	0%
**AEs throughout the treatment cycle**								
Nausea	75%	62%	8%	4%	25%	24%	1%	1%
Vomiting	25%	17%	8%	0%	6%	5%	0%	1%

The denominator for each cycle-specific adverse event represents the number of patients who actually received the corresponding cycle of Zolbetuximab treatment.
