# Supplementary figures and images for: Practical Management of Zolbetuximab Administration: The Project VYLOY Initiative
Source: Cancers (Basel). 2025 Jun 15;17(12):1996. doi: 10.3390/cancers17121996 (PMC12190778; doi:10.3390/cancers17121996)

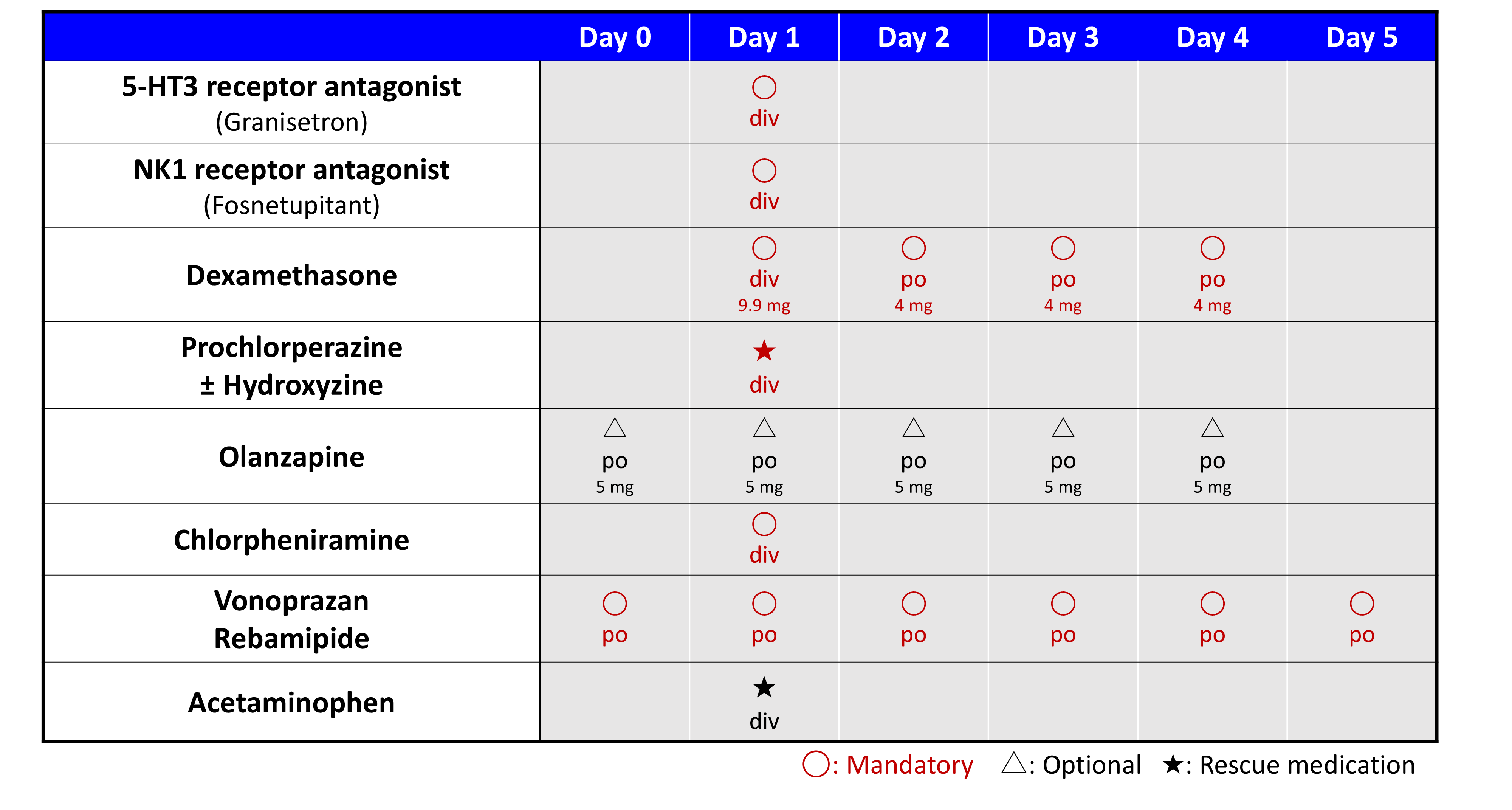

Supplement: Supplementary file 1 [file cancers-17-01996-s001.zip › Supplementary Figure S1.tif]

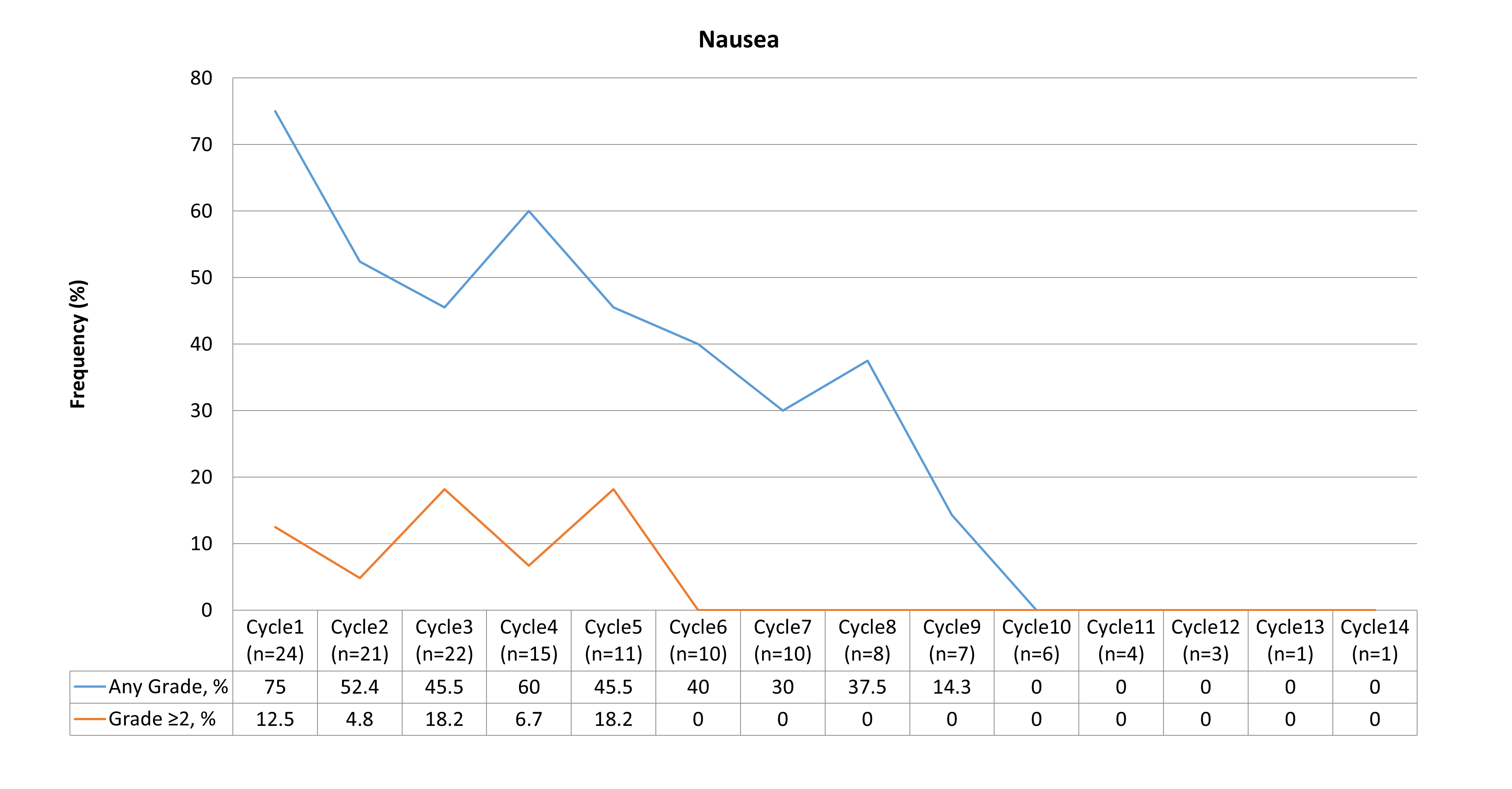

Supplement: Supplementary file 1 [file cancers-17-01996-s001.zip › Supplementary Figure S2A.tif]

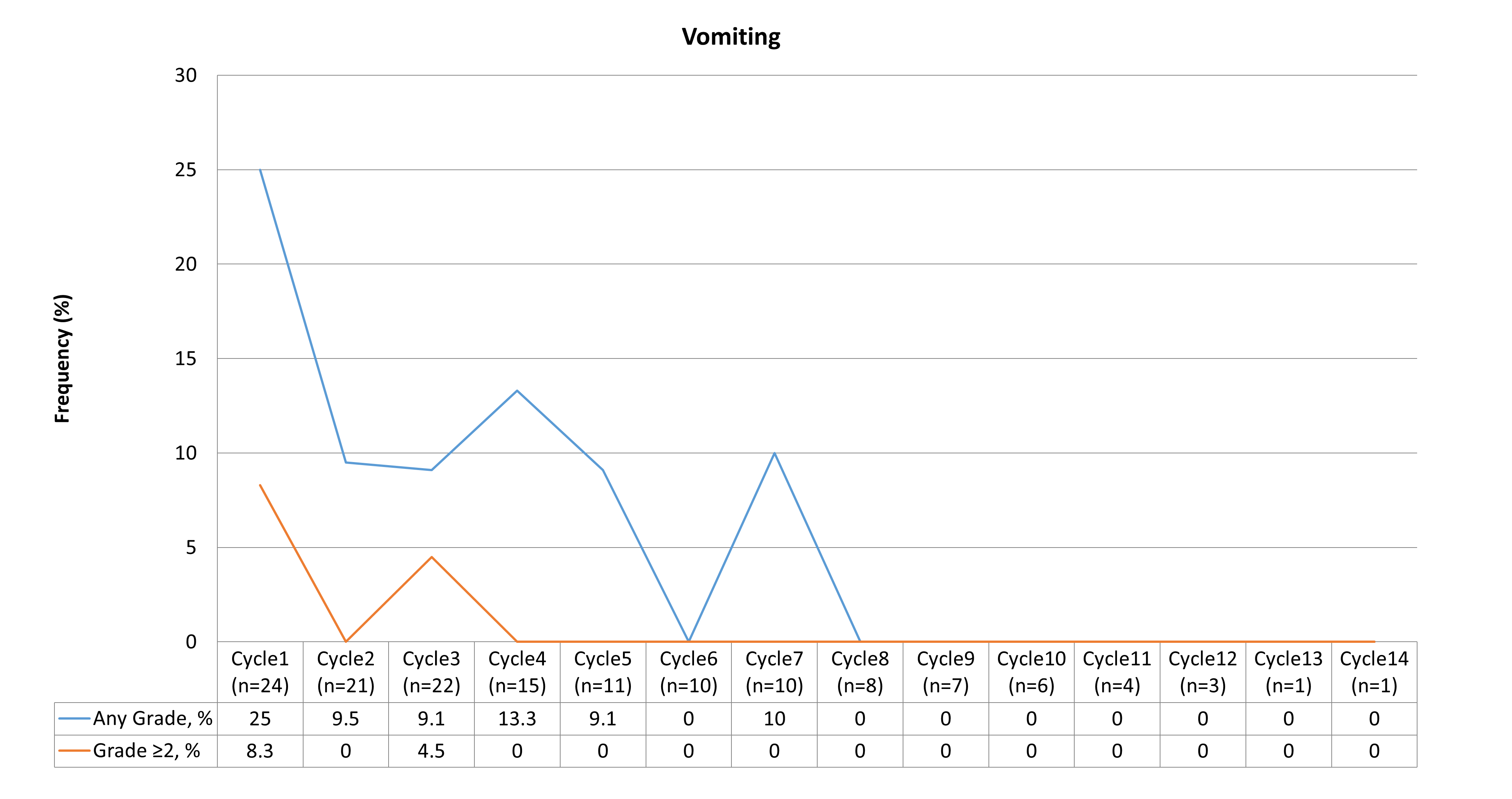

Supplement: Supplementary file 1 [file cancers-17-01996-s001.zip › Supplementary Figure S2B.tif]

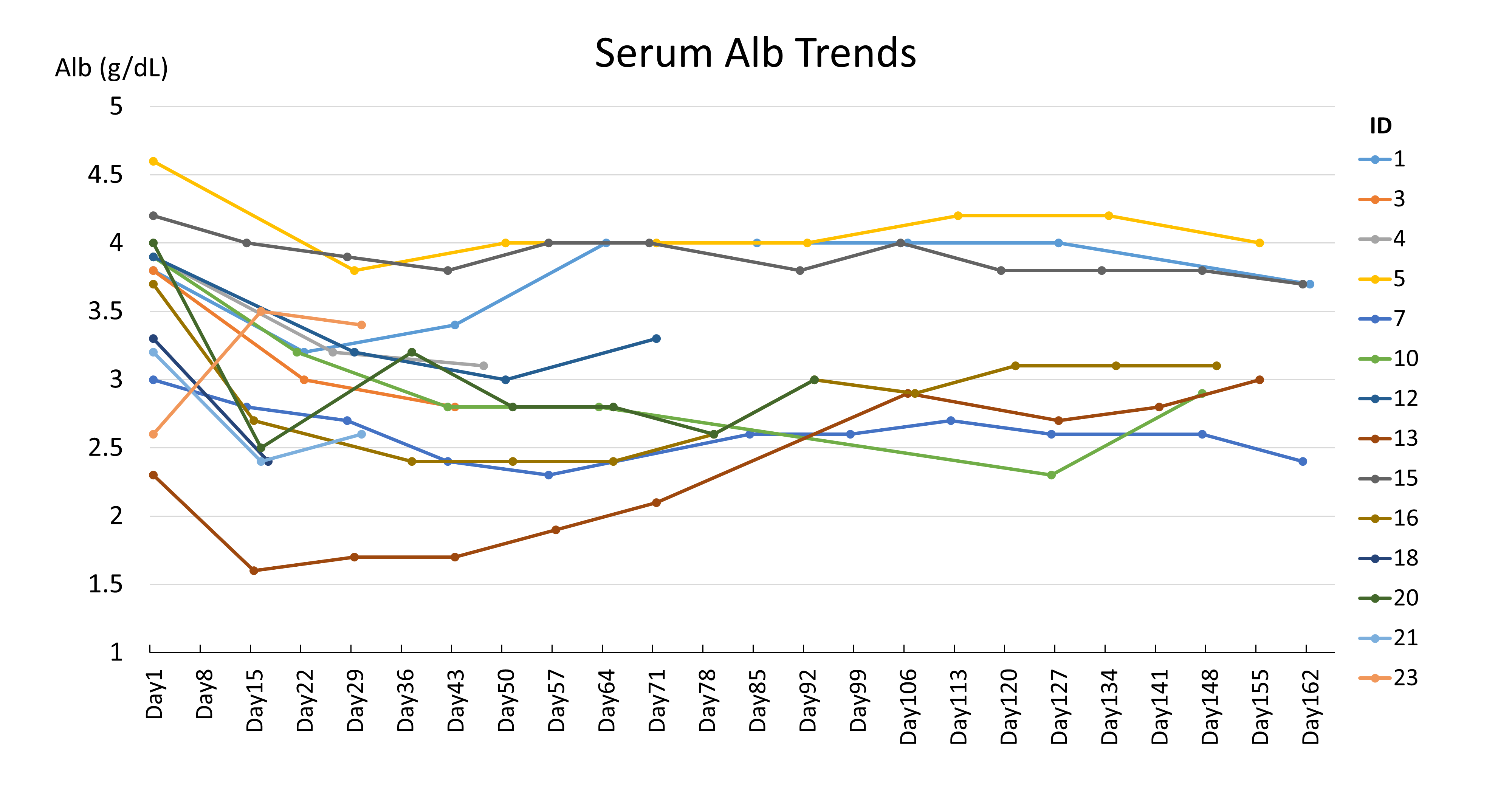

Supplement: Supplementary file 1 [file cancers-17-01996-s001.zip › Supplementary Figure S3.tif]

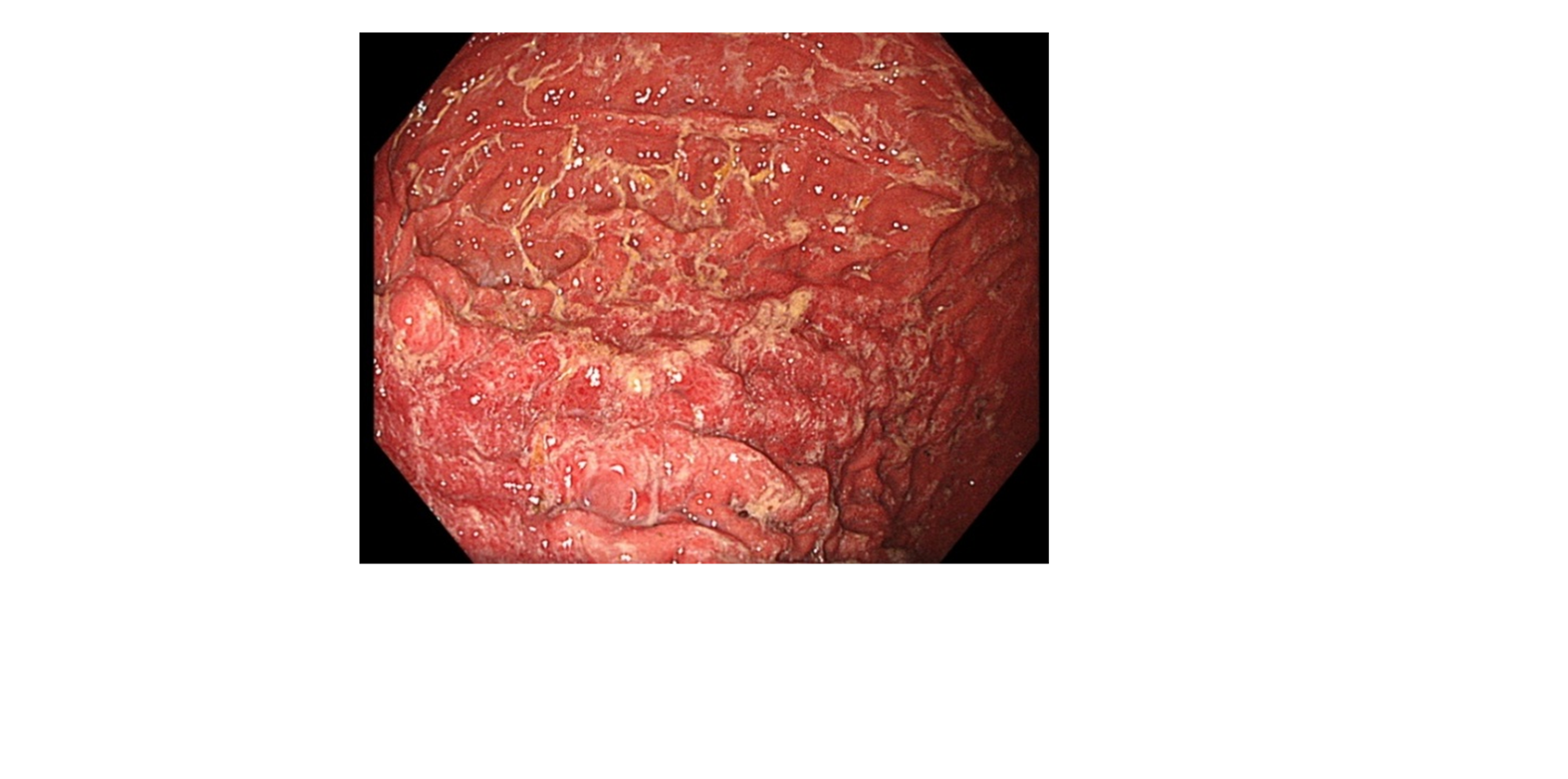

Supplement: Supplementary file 1 [file cancers-17-01996-s001.zip › Supplementary Figure S4A.tif]

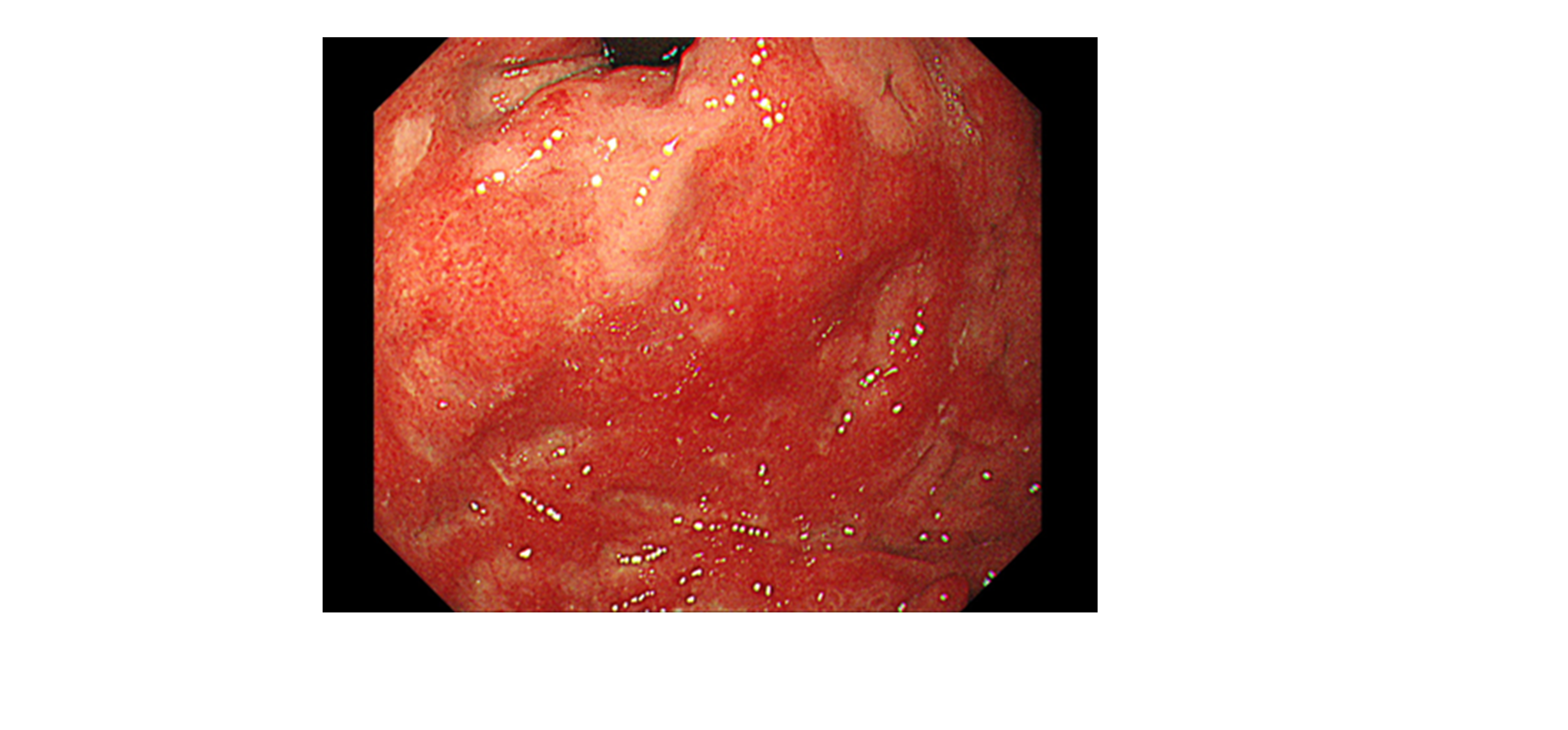

Supplement: Supplementary file 1 [file cancers-17-01996-s001.zip › Supplementary Figure S4B.tif]
